# Seasonal Spatial Distribution Patterns of *Abralia multihamata* in the East China Sea Region: Predictions Under Various Climate Scenarios

**DOI:** 10.3390/ani15070903

**Published:** 2025-03-21

**Authors:** Min Xu, Shuhao Liu, Chunhui Yang, Linlin Yang

**Affiliations:** 1 Key Laboratory of East China Sea Fishery Resources Exploitation, Ministry of Agriculture and Rural Affairs, Shanghai 200090, China; xuminwzy@aliyun.com; 2 East China Sea Fisheries Research Institute, Chinese Academy of Fishery Sciences, Shanghai 200090, China; 3 First Institute of Oceanography, Ministry of Natural Resources, Qingdao 266100, China; lshuhao666@gmail.com; 4 Marine Living Resources and Environment Key Laboratory of Hebei Province, Ocean and Fisheries Science Research Institute of Hebei Province (Marine Fishery Ecological Environment Monitoring Station of Hebei Province), Qinghuangdao 066200, China

**Keywords:** greenhouse gas, pCO_2_, northwest Pacific, aquatic animal, Teuthoidea, carbon sequestration, SST, SSP

## Abstract

The enoploteuthid squid species *Abralia multihamata* plays an important role in the epi- and mesopelagic food web. However, little is known about its seasonal and spatial distribution, life history traits, and environmental threats that may affect it. In this study, we used independent scientific bottom trawling surveys conducted in the southern Yellow and East China Seas during 2018–2019 to identify the seasonal spatial distribution characteristics of biomass, number, and size of this species as well as the relationships among these features and measured environmental factors. We also predicted the habitat distribution variations of the species under different climate scenarios (the present, SSP1–2.6, SSP2–4.5, SSP3–7.0, and SSP5–8.5) and seasons. The results revealed a continuously increasing individual size from the southern Yellow Sea to the East China Sea in spring, summer, and autumn, which suggests the possibility of growth differences in different water temperature conditions. The seasonal order of regional mean biomass and number was autumn > spring > summer and winter in the study area, and, for size, it was spring > summer and autumn > winter. This result shows that the majorities of recruitment and breeding groups occurred in autumn and spring, respectively. In addition, our results showed that the most beneficial case in terms of average habitat area was SSP3–7.0 in 2050, and the most loss occurred under SSP1–2.6 and SSP5–8.5 in 2100. Few benefits were predicted for the other cases under the various climate scenarios. This study provides a new understanding of the distribution and life history of *A. multihamata* in the East China Sea region.

## Simple Summary

Previous studies have suggested that cephalopods might benefit from climate change. In this study, we used the artificial neural network, classification tree analysis, flexible discriminant analysis, generalized additive model, generalized boosting model, generalized linear model, multiple adaptive regression splines, random forest, surface range envelope, and extreme gradient boosting training algorithms to develop ensemble models to forecast the habitat distribution variations of the squid *Abralia multihamata* under different climate scenarios (SSP1–2.6, SSP2–4.5, SSP3–7.0, and SSP5–8.5) and seasons. The results of this study can be applied to fisheries management actions to mitigate the negative effects of climate change.

## 1. Introduction

Since the 1850s, climate changes have increased, and increasing greenhouse gas concentrations result in climate disequilibrium [1]. pCO_2_ in the atmosphere has increased from a preindustrial concentration of 280 ppm to more than 400 ppm [2]. The Intergovernmental Panel on Climate Change estimated that pCO_2_ in the atmosphere could reach 1000 ppm by the end of this century [3]. Climate change can affect aquatic animals at the individual and school levels [4]. Dawe and Warren (1993) suggested that recruitment of the ommastrephid squid *Illex illecebrosus* in the northwest Atlantic might be regulated by climatic variations [5]. Ma et al. (2019) found that decadal variations of fisheries resources in the East China Sea region corresponded with contemporaneous climatic regime shifts in the Pacific, and that different marine organisms have diverse sensitivities to patterns of climate-induced change [6]. Sea bottom organisms, including cephalopods that live near the benthos, are thought to be more vulnerable to climate changes [7].

Cephalopods are nektonic marine mollusks [8]. They are characterized by bilateral body symmetry, a prominent head, and a set of arms or tentacles modified from the primitive molluscan foot [8]. Squid play crucial roles in marine ecosystems, as both predators and prey. The life expectancy of squid is generally about 1–2 years, and individuals of most species die after spawning [9].

Enoploteuthid squids, the ‘myctophid fishes’ of the squid world, generally inhabit middle depths and continental shelves [10] They play a significant role in both epipelagic and mesopelagic trophic webs [11,12]. The genus *Abralia* contains 20 nominal species, and they are small species associated with shallow bottoms [13]. These species are members of the mesopelagic-boundary fauna and are key members of the micronektonic groups in the tropical and subtropical oceans [14]. Guerra-Marrero et al. (2020) suggested the possibility that *Abralia veranyi* and *Abraliopsis morisii* may play a key role in the oceanic food web [15]. According to the IUCN Red List of threatened species, *Abralia multihamata* (Coleoidea Teuthoidea Enoploteuthidae) is a species of least-concern, and little is known about its seasonal-spatial distribution, life history, resource status, and environmental threats that may affect it. As noted by Li et al. (2006), this species is rarely studied in the East China Sea region [16].

Squid are generally believed to be unstable and strongly affected by environmental conditions [17], and they have been used as biological indicators of environmental variations such as sea surface temperature (SST) changes in the oceans [18]. The young stages of squids are particularly sensitive to environmental change [19]. Pierce and Boyle (2003) reported that recruitment strength of cephalopods is often related to water temperatures during the early life history stages [20]. Additionally, studies have suggested that landings of many squid species are closely related to seawater temperature [21]. It is necessary to identify the relationship between the environmental variables and population characteristics for *A. multihamata*.

The species distribution model is a model based on niche theory and widely used to analyze the spatiotemporal distribution of marine organisms. It is extensively applied to the research on cephalopods [22,23,24]. The following models are commonly used in such research. The generalized linear model (GLM), is often used to express the relationship between environmental factors and modeling objectives through regression equations. It is suitable for response variables that are numerical, with good explanatory power. The generalized additive model (GAM), building on GLM, can handle nonlinear relationships among variables. Artificial neural networks (ANNs), which simulate the structure and function of human brain neurons, can process complex nonlinear relationships with high prediction accuracy. The maximum entropy model (MaxEnt) is based on the principle of maximum entropy and used to estimate species distribution probabilities by maximizing entropy. It requires less data and is capable of handling environmental factors with nonlinear relationships. Random forest (RF) is an ensemble learning method based on decision trees that can process high-dimensional data and has good generalization ability.

In this study, we aimed to identify the seasonal spatial distribution characteristics of biomass, number, and size of *A. multihamata* in the East China Sea region. We assessed the relationship between seasonal variations in number of *A. multihamata* and measured environmental factors. Finally, we predicted the seasonal distribution characteristics of *A. multihamata*, and their annual yearly presence under climate scenarios (Shared Socioeconomic Pathway (SSP1–2.6, SSP2–4.5, SSP3–7.0, and SSP5–8.5) in 2050 and 2100. For these scenarios, we estimated the loss%, gain%, and gain%–loss% of habitat areas of *A. multihamata*. Our results provide a better understanding of the relationship between the population dynamics of *A*. *multihamata* and the environment, which might enable researchers to forecast these fluctuations and managers to regulate fishing efforts to provide ecosystem and social benefits.

## 2. Materials and Methods

### 2.1. Sampling and Survey Procedures

**Figure 1:**
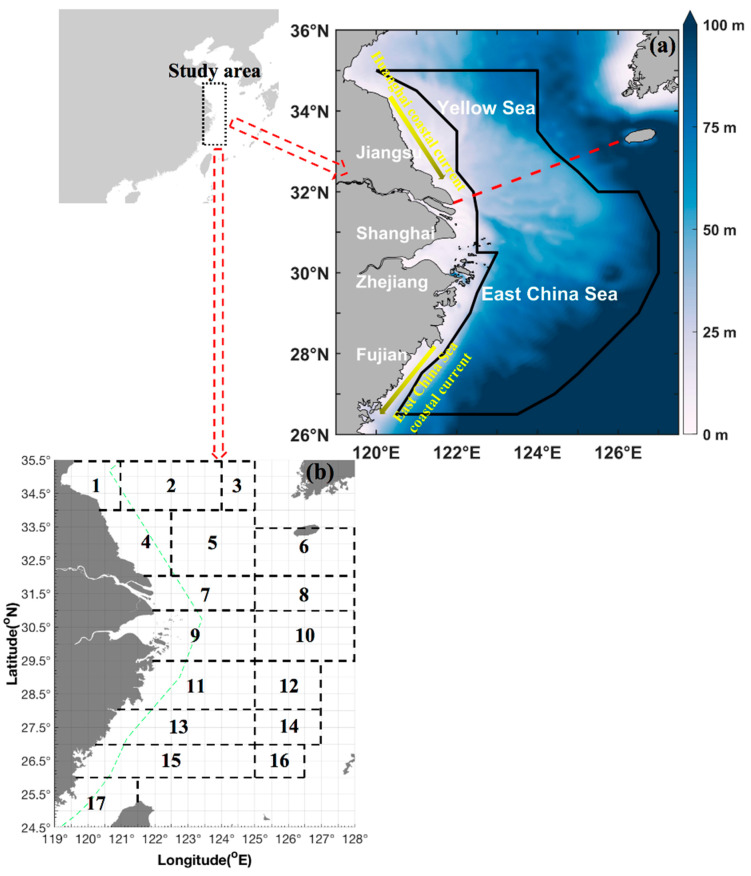
(**a**) Map of the study area (26.50–35.00° N 120.00–127.00° E), which is denoted by a dark blue solid line in the East China Sea region, including the Southern Yellow and East China Seas adjacent to the coastline of Fujian, Zhejiang, Shanghai, and Jiangsu. The color bar denotes the depth range from 0 m to 100 m. The red dashed line indicates the boundary between the Yellow and East China Seas. The yellow arrows indicate the Huanghai coastal current and the East China Sea coastal current. (**b**) The black boxes and numbers represent the following fishing grounds: (1) Haizhou Bay, (2) Lianqingshi, (3) Liandong, (4) Lvsi, (5) Dasha, (6) Shawai, (7) Yangtze river mouth, (8) Jiangwai, (9) Zhoushan, (10) Zhouwai, (11) Yushan, (12) Yuwai, (13) Wentai, (14) Wenwai, (15) Mindong, (16) Minwai, and (17) Minzhong. The green dashed line marks the motor-trawl prohibition line.

This study did not involve endangered or protected species listed in the China Red Data Book of Endangered Animals. Independent scientific bottom trawling surveys were conducted in the southern Yellow and East China Seas (also called as East China Sea region) during 2018 and 2019. The surveys used a trawl net with a cod end mesh size of 20 mm and a height of 10–15 m that was towed by fisheries research vessels (the Zhongkeyu 211 and 212) in autumn (2–11 November 2018: 48,293.27 g·h^−1^ of total catch per unit effort by weight (CPUE_w_) and 15,152 ind·h^−1^ of total catch per unit effort by number (CPUE_n_)), winter (4–27 January 2019: 8543.98 g·h^−1^ of total CPUE_w_ and 2482 ind·h^−1^ of total CPUE_n_), spring (22 April–10 May 2019: 15,225.55 g·h^−1^ of total CPUE_w_ and 3288 ind·h^−1^ of total CPUE_n_), and summer (13 August–27 September 2019: 5474.4 g·h^−1^ of total CPUE_w_ and 1314 ind·h^−1^ of total CPUE_n_). The study area covered 26.50–35.00° N, 120.00–127.00° E (Figure 1). The survey stations were determined using a sampling grid with dimensions of 30 min of latitude and 30 min of longitude (30′ × 30′). The average trawl speed was 3 knots and all tows were conducted for approximately 1 h at each station. In total, 519 valid tows were included in this study: 127 stations in autumn, 111 stations in winter, 141 stations in spring, and 140 stations in summer.

The catches were analyzed in the laboratory to identify the species caught and assess their occurrence at each station. The total sample of *A. multihamata* in each station was counted and weighed to the nearest 0.10 g of wet weight, and the catch density of this species was calculated as biomass density per unit of sampling time (g·h^−1^) and individual numerical density per unit of sampling time (ind·h^−1^). The average individual weight (AIW) was defined as the CPUE_w_ divided by the CPUE_n_ at each station. Environmental variables, including water depth, water temperature, salinity, and DO concentration, were measured at each station using a conductivity–temperature–depth profiler (SBE-19; SeaBird-Scientific, Bellevue, WA, USA). SST (sea surface temperature), SSS (sea surface salinity), and SSDO (sea surface dissolved oxygen) were measured at 3 m below the surface, and SBT (sea bottom temperature), SBS (sea bottom salinity), and SBDO (sea bottom dissolved oxygen) were measured 2 m above the sea bottom at sea depths < 50 m) and at 2–4 m above the bottom at sea depths > 50 m (Figure S1).

### 2.2. Ensemble Model, Selection of Environmental Variables, and Calibrations

Species distribution models (SDM) are extensively applied to identify the habitat distribution variations of cephalopods [22,23,24]. In this study, we used the species distribution model to describe and forecast the relationship between *A. multihamata* and environmental variables. SDM has been widely applied to forecasting the habitat distribution of marine animals in China’s Seas and other sea areas [25,26,27]. We used the following 10 algorithms to predict the habitat distribution of *A. multihamata*: artificial neural network (ANN), classification tree analysis (CTA), flexible discriminant analysis (FDA), generalized additive model (GAM), generalized boosting model (GBM), generalized linear model (GLM), multiple adaptive regression splines (MARS), random forest (RF), surface range envelope (SRE), and extreme gradient boosting training (XGBOOST). Araujo and New (2007) reported that the ensemble model that combines these 10 models was more advantageous compared with single models, and this can largely improve the robustness of the prediction and decrease analysis bias, thereby yielding more confidence in the predictions [28,29].

We used the “biomod2” package in the ensemble SDM planform [30]. To run the model, the data set was separated into categories of 0 (absence) and 1 (presence), and an 80%:20% split was then randomly applied for training and testing data independently to construct the 10 algorithms using the random cross-validation method [31]. Each algorithm was run 20 times to obtain 200 models to obtain stable results. We used the mean survey data over four months to produce the annual model, and used different seasonal data to produce the seasonal models. All the data used in the models were obtained from surveys conducted as part of this study. The performance of each algorithm was assessed by the index of the area under the receiver operating characteristic curve (ROC) and the true skill statistic (TSS). Among these 200 models, we selected those that performed best (a threshold value of the receiver operating characteristic curve (AUC) > 0.8) and combined them into an ensemble model using the weighted average method [30]. The computation code used is detailed elsewhere [32]. Details regarding the function and usage of variable importance can be found at (https://biomodhub.github.io/biomod2/reference/bm_VariablesImportance.html, accessed on 16 March 2025).

Future climate data were obtained from the Coupled Model Intercomparison Project Phase 6 (CMIP6) [33], and environmental data such as SST, SBT, SSS, and SBS, were obtained from the website Bio-ORACLE: marine data layers for ecological modeling (https://bio-oracle.org/index.php, accessed on 16 March 2025). The four Shared Socioeconomic Pathways (SSPs) scenarios (SSP1–2.6, SSP2–4.5, SSP3–7.0, and SSP5–8.5) for 2040–2050 (the 2050s) and 2090–2100 (the 2090s) were used in this study. The SSP1–2.6 scenario is a sustainable development situation that emphasizes sustainability, low resource consumption, and low carbon emissions. The SSP2–4.5 scenario is the intermediate challenges scenario, with a radiative stabilization rate of 4.5 W m^−2^ beyond 2100. The SSP3–7.0 scenario contains a medium to high forcing regional rivalry pathway with a radiative stabilization rate of 7.0 W m^−2^. Finally, the SSP5–8.5 scenario encompasses a fossil fuel-driven development situation characterized by high carbon emissions and assumes that future societies will rely heavily on fossil fuels to power economic growth [34].

Bias corrections were performed for SST, SSS, SBT, and SBS. Climate models, while foundational, possess intrinsic limitations that can introduce biases in projected environmental variables [35]. These biases have the potential to compromise the precision of species distribution models. Bias correction of climate model raw data is essential to enhance the credibility of habitat distributions under future climate scenarios [36]. The delta method is a prevalent technique in fisheries habitat prediction that effectively mitigates such biases [37]. We employed this approach to calculate climate differences between contemporary and future datasets by applying corrections to raw data. Specifically, the delta method leverages discrepancies between observed and simulated baseline conditions to adjust simulations for time (*t*) periods (2040–2050 and 2090–2100).

Bias correction for time *t* in geographical location x was conducted as follows:

$$D_{\mathrm{sim}}^{\mathrm{DM}}\left(x,\;t\right)=D_{\mathrm{emp}}\left(x,0\right)+\left(D_{\mathrm{sim}}^{\mathrm{raw}}\left(x,\;t\right)-D_{\mathrm{sim}}^{\mathrm{raw}}\left(x,\;0\right)\right)\;=D_{\mathrm{sim}}^{\mathrm{raw}}\left(x,\;t\right)+\left(D_{\mathrm{emp}}\left(x,0\right)-D_{\mathrm{sim}}^{\mathrm{raw}}\left(x,\;0\right)\right)$$

where $D_{\mathrm{emp}}\left(x,0\right)-D_{\mathrm{sim}}^{\mathrm{raw}}\left(x,\;0\right)$ represents the bias as the anomaly between observed and simulated environmental data at location *x*, and $D_{\mathrm{sim}}^{\mathrm{DM}}\left(x,\;t\right)$ denotes the bias-corrected temperature forecasts that were calculated by adding the bias to the simulated environmental data for time *t* in geographical location *x*.

## 3. Results

### 3.1. Seasonal Spatial Distribution Characteristics of Biomass, Number, and Size

**Figure 2:**
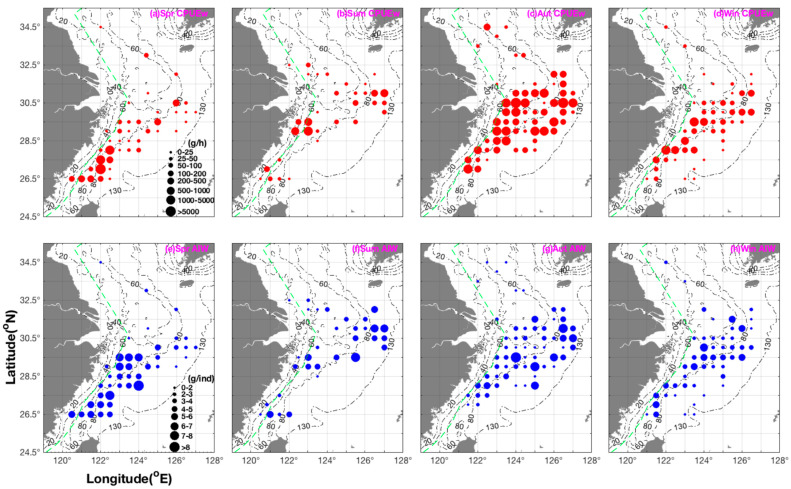
Seasonal distribution patterns of catch per unit effort by weight (g·h^−1^), shown in red (grouped into 0–25, 25–50, 50–100, 100–200, 200–500, 500–1000, 1000–5000, and >5000 g·h^−1^), and AIW (g·ind^−1^) shown in blue (grouped into 0–2, 2–3, 3–4, 4–5, 5–6, 6–7, 7–8, and >8 g·ind^−1^) for *Abralia multihamata*. The values are represented by filled circle size. The depth gradient (20–130 m) is represented by a black dash-dot line. The green dashed line indicates the closed fishing lines, which pertains to the boundary of areas in which fishing is forbidden to prevent the destruction of aquatic resources by wheel trawling.

In spring, the squid exhibited a scattered distribution in the southern Yellow Sea of China within the spatial range (fishing grounds of Changjiangkou, Lusi, and Dasha). We found larger individuals in the fishing grounds of Wentai and Mindong. The regional mean biomass in the Wentai fishing ground was greater than that of the Zhoushan fishing ground. The mean size continuously increased from the southern Yellow Sea to the northern and central sections of the East China Sea. In addition, larger and smaller individuals, respectively, were found in the areas of 124.00° E to the west and 124.00° E to the east, with most of the biomass in inshore waters (Figure 1 and Figure 2).

The data collected during the summer assumed the presence of a possible nursery ground at the central and southern section of the Zhoushan fishing ground, and the majority of juveniles were found in the Wentai fishing ground. The regional mean biomass in the Yushan fishing ground was greater than that of the Zhoushan fishing ground. The mean size increased in the latitudinal order of 32.50° N→32.00° N→31.50° N→31.00° N→30.50° N (fishing grounds of Changjiangkou and Zhoushan). Generally, in this season, the greatest numbers of individuals and biomass were found in the middle part of the coastal fishing grounds in the study area, with larger and smaller individuals, respectively, in the longitudinal ranges of 125.00° E–127.00° E and 120.50° E–125.00° E (Figs 1 and 2).

In autumn, the latitudinal mean biomass was present in the following order of fishing grounds: middle Zhoushan > middle and northern part of Yushan > northern Zhoushan. The latitudinal mean size increased in the order of 33.50° N–34.50° N→32.00° N–33.00° N→31.50° N→31.00° N→30.50° N. The greatest biomass was found in the longitudinal range of 123.00° E–124.00° E and 125.50° E–126.50° E. In winter, the majority of juvenile squid were found in the fishing grounds from Lusi to Haizhou Bay. In terms of latitudinal range, the largest biomass was found in the Yushan fishing ground; for longitudinal range, the largest biomass was found in 122.00–123.50° E, with the largest individual sizes at 126.00° E (Figure 1 and Figure 2).

The mean CPUE_w_ and CPUE_n_ values in the study area were in the order of autumn > spring > summer and winter, and the mean AIW values were in the order of spring > summer and autumn > winter. The highest values of both CPUE_w_ and CPUE_n_ were in the order of spring > autumn > winter > summer. The highest and lowest values of AIW were in the order of spring > summer and autumn > winter, indicating the decreasing mean individual size from spring to winter (Table 1).

### 3.2. Seasonal Variations of Measured Environmental Factors

**Figure 3:**
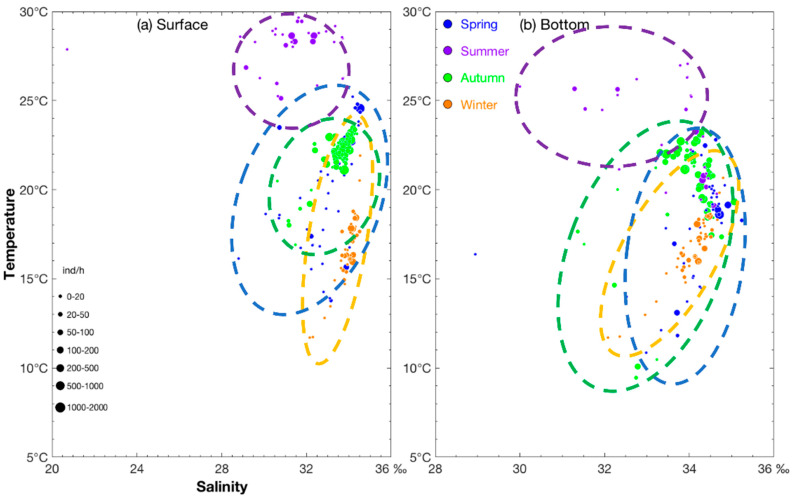
Relationship between salinity (‰) and temperature (°C) for catch per unit effort by number (CPUE_n_) sizes classified by group (0–20, 20–50, 50–100, 100–200, 200–500, 500–1000, and 1000–2000 ind·h^−1^) and average individual weight (AIW) sizes classified by group (0–1, 1–2, >6 g·ind^−1^) of *Abralia multihamata*. The data for spring, summer, autumn, and winter are denoted by solid blue, purple, green, and brown–red circles, respectively. (**a**) sea bottom temperature vs. sea bottom salinity for CPUE_n_; (**b**) sea surface temperature vs. sea surface salinity for CPUE_n_.

*A. multihamata* was distributed in shallow waters from spring to autumn but migrated to deeper areas in winter (Table 1). Most *A. multihamata* individuals were found in SST of 22.68–24.56 °C, SBT of 18.6–19.13 °C, SSS of 33.87–34.52‰, and SBS of 34.68–34.91‰ in spring; SST of 28.31–28.65 °C, SSS of 31.31–32.36‰, the locations of SBT 23.27 °C and SBS 34.15‰ and SBT 19.08–20.78 °C and SBS 34.32–34.59‰ in summer; SST of 21.1–23.22 °C, SBT of 20.8–21.99 °C, SSS of 32.96–34.19‰, and SBS of 33.46–34.48‰ in autumn; and SST of 15.94–18.41 °C, SBT of 15.97–18.52 °C, SSS of 34.1–34.34‰, and SBS of 34.19–34.48‰ in winter (Figure 3a,b). The seasonal SST values showed that the species had a wide water temperature range but a preference for high water temperature from summer to winter. With reference to SSS, from spring to summer *A. multihamata* migrated to inshore areas; they moved to high and higher SSS areas, respectively, in autumn and winter, and then to low SSS areas from winter to spring. The seasonal SSDO and SBDO values were 8–9 mg L^−1^ in spring, within a wide range at the surface and very low at the bottom in summer, and 7–9 mg L^−1^ in winter (Table 1).

### 3.3. Calibration of Algorithms, and Predictions Under Climate Scenarios

In the algorithms of CTA, FDA, GBM, GLM, MARS, RF, XGBOOST, and ensemble models, variability in SBS was most important. In the algorithms of ANN and SRE, SBT was most important, and, in the GAM algorithm, SSS variability was most important (Figure S2). In terms of the TSS and ROC, RF was the best model and SRE had the worst fit (Figures S3 and S4).

They showed the tendency of inshore expansion from spring to winter in the southern East China Sea (Figures S5 and S6). Under the SSP1–2.6, SSP2–4.5, SSP3–7.0, and SSP5–8.5 climate scenarios, the distribution habitat was predicted to move offshore from 2050 to 2100 (Figures S7 and S8). The climate scenarios with the greatest predicted habitat area loss percentage were SSP5–8.5 > SSP3–7.0 > SSP1–2.6 in 2100. The climate scenarios with the greatest habitat area gain percentage were SSP3–7.0 in 2050 > SSP3–7.0 in 2100 > SSP2–4.5 in 2100. For the case of Gain% − Loss%, the best scenario was SSP3–7.0 in 2050, whereas the most negative cases were SSP1–2.6 and SSP5–8.5 in 2100 (Table 2).

## 4. Discussion

Generally, *Abralia* spp. are deep-water marine animals that are distributed worldwide and that spawn in coastal areas [38]. Sasaki et al. (1914) reported that *Abralia watasenia* survives at depths > 100 m [39]. *A. veranyi* mainly survives in the upper layers of the mesopelagic zone with from 200 to 500 m depth [40]. At night, *A. veranyi* was recorded at depths as shallow as from 38 to 90 m, whereas *Abraliopsis morisii* was found at depths of from 98 to 219 m [15]. Statolith data from *Abralia trigonura* showed an ontogenetic shift from an epipelagic to a mesopelagic habitat, with adults occupying the mesopelagic habitat [41]. Silas (1968) proposed that *Abralia andamanica* might be distributed along the continental shelf edge in the Arabian Sea [42]. In the current study, *A. multihamata* was widely distributed in the study area within a depth range of 16 to 145 m. However, most of the biomass and numbers were in inshore areas from spring to summer, indicating the potential presence of spawning and nursery grounds. We propose that *A. multihamata* moves to shallower inshore areas to spawn and grow in spring to summer and then migrates to offshore areas in autumn and winter for overwintering. We suggest that the nursery grounds of *A. multihamata* include the fishing grounds of central and southern Wentai and the central part of Mindong in spring, the central and southern section of the Zhoushan fishing ground in summer, 30.00–27.00° N in the southern East China Sea in autumn, and the fishing grounds of Lusi to Haizhou Bay in winter.

Regarding migration and distribution, Cabanellas-Reboredo et al. (2014) reported that European squid migrate from deeper to inshore waters [43]; this enables the hatchlings to grow by taking advantage of abundant prey organisms [44]. For paralarvae produced during this period, the embryonic phase is associated with a gradual warming of water temperature [45]. Among the environmental factors that affect squid, seawater temperature plays a key role in variations in squid abundance. Moriwaki (1994) found a close relationship between the cold water mass at the sea bottom and the migration of the swordtip squid *Photololigo edulis* [46]. Young and Mangold (1994) identified the importance of SST during spawning activities of *A. trigonura* [47], and Miyahara et al. (2005) found a positive relationship between recruitment of *Thysanoteuthis rhombus* in the western Sea of Japan from September to October and seawater temperatures 600 km upstream in the Tsushima Strait in June [48]. In our study of *A. multihamata*, paralarvae and juveniles had enough food in the near-shore areas; they preferred the high water temperature environment, which reinforced their potential for rapid growth.

In general, studies have suggested that cephalopods may benefit from climate change. Currently, the distribution of coastal species has been either observed [49] or projected [50,51] to shift poleward or into deeper waters [52] under climate change scenarios. The species *Doratosepion braggi* (Verco, 1907) could decline by as much as 30.77% in average habitat suitability from the present of 55.26% to 24.48% at SSP5–8.5 in 2100, and *Sepia officinalis* (Linnaeus, 1758) showed a low maximum decrease of 1.64% in average habitat suitability from the present of 59.62% to 57.98% at SSP5–8.5 in 2100 [7]. The current habitat area range of *Sepia esculenta* was found to be more northerly than previously report [53]. By the 2050s, the joint distribution areas of *Loliolus beka* and *Loliolus uyii* will have gradually expanded to the central East China Sea and the southern Yellow Sea; by the 2090s, the distribution area of both species will have significantly reduced in the coastal areas of the southern Yellow Sea and middle areas of East China Sea [54]. The habitat area of *Sepiella maindroni* will first expand and then greatly decrease with the intensification of global warming [55]. The habitat area of *Sepia kobiensis* will be increasing [55]. The core habitat of *AmphiOctopus ovulum* is expected to expand to the northeast and southwest independently [56]. The annual mean habitat area of *AmphiOctopus fangsiao* will shrink significantly under the SSP5–5.8 scenario by 2050 and by 2100 [57]. The annual mean habitat of *Octopus variabilis* will shift northward offshore under SSP1–2.6 scenario by 2050 and by 2100, but will shrink significantly under SSP5–8.5 by 2100 [57]. Our scenarios also suggest that *A. multihamata* moves poleward from spring to winter. Overall, our comparison of the current scenario with the results produced by other predictable models can be used to inform fisheries management and policy decisions under climate variability and change. Our study has some limitations. Specifically, the methods used in this study are subject to a risk of overfitting in predicting future distributions of the species under different climate scenarios [58].

## 5. Conclusions

In this study, we identified seasonal spatial distribution characteristics of *A. multihamata* and their relationships with environmental variables in the southern Yellow and East China Seas. They showed the tendency of inshore expansion from spring to winter in the southern East China Sea. Future studies should focus on identifying the distribution of prey organisms and analyzing the relationships between *A. multihamata* and its prey. Additionally, a better understanding that integrates social, economic, and ecological considerations is needed of the changes in the distribution and migration patterns and routes of *A. multihamata* under different climate scenarios. We appeal that ocean governance should consider climate change and forecasting to drive transformative, sustainable, and inclusive ocean care.

## Figures and Tables

**Table 1:** Seasonal data ranges of environmental factors (depth, SST, SBT, SSS, SBS, SSDO, SBDO) in situ; biological indicators (mean CPUE_w_ at collection stations, value range of CPUE_w_, mean CPUE_n_ at collection stations, value range of CPUE_n_, mean AIW, value range of AIW) in the study area from autumn 2018 to summer 2019 ^a^.

Factor	Spring	Summer	Autumn	Winter
Depth (m)	23.00–118.00	16.00–97.00	21.00–107.00	39.00–145.00
SST (°C)	13.77–25.23	25.14–29.67	16.91–23.66	11.68–23.71
SBT (°C)	10.85–22.79	18.87–27.06	9.47–23.15	11.68–20.67
SSS (‰)	28.80–34.53	20.72–33.74	30.64–34.38	32.16–34.55
SBS (‰)	28.95–35.25	30.00–34.65	31.37–35.07	32.08–34.80
SSDO (mg/L)	7.84–8.43	4.51–10.53	/	6.92–8.62
SBDO (mg/L)	7.76–8.98	2.73–6.60	/	7.30–8.58
Mean CPUE_w_ at collection stations (g/h)	362.55	171.08	791.69	174.37
Value range of CPUE_w_ (g/h)	1.34–10,184.0	10.80–1405.76	1.62–6064.59	0.60–1914.40
Mean CPUE_n_ at collection stations (ind/h)	78.31	41.06	248.39	50.65
Value range of CPUE_n_ (ind/h)	1.00–1920.00	3.00–285.00	1.00–1764.00	1.00–496.00
Mean AIW (g/ind)	3.99	3.71	3.64	3.04
Value range of AIW (g/ind)	1.16–8.34	1.20–7.80	0.90–8.00	0.60–6.50

^a^ Abbreviations: SST, sea surface temperature; SBT, sea bottom temperature; SSS, sea surface salinity; SBS, sea bottom salinity; SSDO, sea surface dissolved oxygen; SBDO, sea bottom dissolved oxygen; CPUE_w_, catch per unit effort by weight; CPUE_n_, catch per unit effort by number; AIW, average individual weight.

**Table 2:** Percentage (%) of habitat loss, gain, and variation (Gain% − Loss%) in cases of the SSP126–2050, SSP126–2100, SSP245–2050, SSP245–2100, SSP370–2050, SSP370–2100, SSP585–2050, and SSP585–2100. The habitat loss and gain are described against the current scenario.

Case	Loss%	Gain%	Gain% − Loss%
SSP126–2050	−2.89%	3.61%	0.72%
SSP126–2100	−5.56%	2.60%	−2.96%
SSP245–2050	−1.87%	3.59%	1.72%
SSP245–2100	−4.09%	9.15%	5.06%
SSP370–2050	−0.77%	10.50%	9.72%
SSP370–2100	−6.79%	9.69%	2.90%
SSP585–2050	−1.96%	3.97%	2.00%
SSP585–2100	−16.95%	7.81%	−9.14%

## Data Availability

The datasets used and/or analyzed during the current study are available from the corresponding author on reasonable request.

## Supplementary Materials

The following supporting information can be downloaded at: https://www.mdpi.com/article/10.3390/ani15070903/s1, Figure S1. Contour map of measured environmental variables including sea surface temperature (SST), sea surface salinity (SSS), sea bottom temperature (SBT), and sea bottom salinity (SBS). Figure S2. Box plots of the importance of environmental variables, including sea bottom salinity (SBS), sea bottom temperature (SBT), sea surface salinity (SSS), and sea surface temperature (SST), in the (a) artificial neural network (ANN), (b) classification tree analysis (CTA), (c) flexible discriminant analysis (FDA), (d) generalized additive model (GAM), (e) generalized boosting model (GBM), (f) generalized linear model (GLM), (g) multiple adaptive regression splines (MARS), (h) random forest (RF), (i) surface range envelope (SRE), (j) extreme gradient boosting training (XGBOOST), and (k) ten ensemble models. Figure S3. Calibration percentage (%) of TSS and ROC in the artificial neural network (ANN), classification tree analysis (CTA), flexible discriminant analysis (FDA), generalized additive model (GAM), generalized boosting model (GBM), generalized linear model (GLM), multiple adaptive regression splines (MARS), random forest (RF), surface range envelope (SRE), and extreme gradient boosting training (XGBOOST) methods. Figure S4. Ratio values of TSS vs. ROC with x-direction and y-direction error bars produced by the artificial neural network (ANN), classification tree analysis (CTA), flexible discriminant analysis (FDA), generalized additive model (GAM), generalized boosting model (GBM), generalized linear model (GLM), multiple adaptive regression splines (MARS), random forest (RF), surface range envelope (SRE), and extreme gradient boosting training (XGBOOST) methods, which are shown in black, blue, green, cyan, red, pink, dark yellow, dark blue, purple, and red-brown, respectively. Figure S5. Spatial distribution patterns of *Abralia multihamata* in the study area predicted with the ensemble model consisting of the artificial neural network, classification tree analysis, flexible discriminant analysis, generalized additive model, generalized boosting model, generalized linear model, multiple adaptive regression splines, random forest, surface range envelope, and extreme gradient boosting training methods from spring to winter (a–d). The color change from blue to green indicates the range from low to high suitability. Figure S6. The predicted habitat suitability in different seasons (spring, summer, autumn, winter). The red and blue area indicate the suitability index of 0.7–1 and 0–0.7 independently. Figure S7. Predicted spatial habitat distribution patterns of *Abralia multihamata* in the cases of (a) annual mean habitat; (b) SSP1–2.6 in 2050; (c) SSP1–2.6 in 2100; (d) SSP2–4.5 in 2050; (e) SSP3–7.0 in 2050; (f) SSP5–8.5 in 2050; (g) SSP2–4.5 in 2100; (h) SSP3–7.0 in 2100; and (i) SSP5–8.5 in 2100. The color change from blue to green indicates the range from low to high suitability. Figure S8. The predicted habitat suitability in different climate scenarios (a) current; (b) SSP1–2.6 in 2050; (c) SSP1–2.6 in 2100; (d) SSP2–4.5 in 2050; (e) SSP2–4.5 in 2100; (f) SSP3–7.0 in 2050; (g) SSP3–7.0 in 2100; (h) SSP5–8.5 in 2050; and (i) SSP5–8.5 in 2100. The red and blue area indicates the suitability index of 0.7–1 and 0–0.7 independently.
