# Supplementary material for: Seasonal Spatial Distribution Patterns of Abralia multihamata in the East China Sea Region: Predictions Under Various Climate Scenarios
Source: Animals (Basel). 2025 Mar 21;15(7):903. doi: 10.3390/ani15070903 (PMC11988177; doi:10.3390/ani15070903)
Supplement: Supplementary file 1 [file animals-15-00903-s001.zip › animals-3493440-supplementary.pdf]

Supplementary file S1

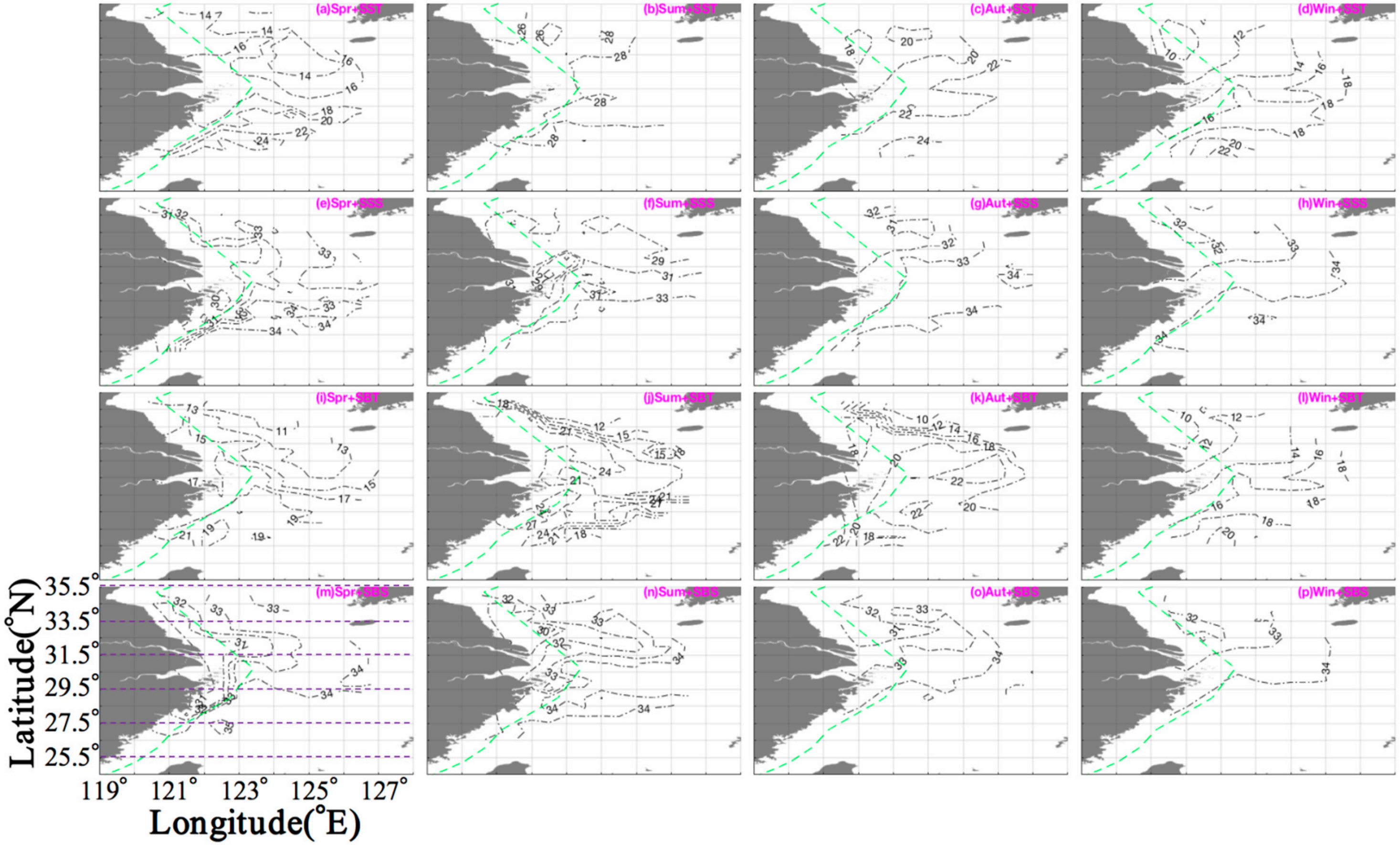

Figure S1. Contour map of measured environmental variables including sea surface temperature (SST), sea surface salinity (SSS), sea bottom temperature (SBT), and sea bottom salinity (SBS).

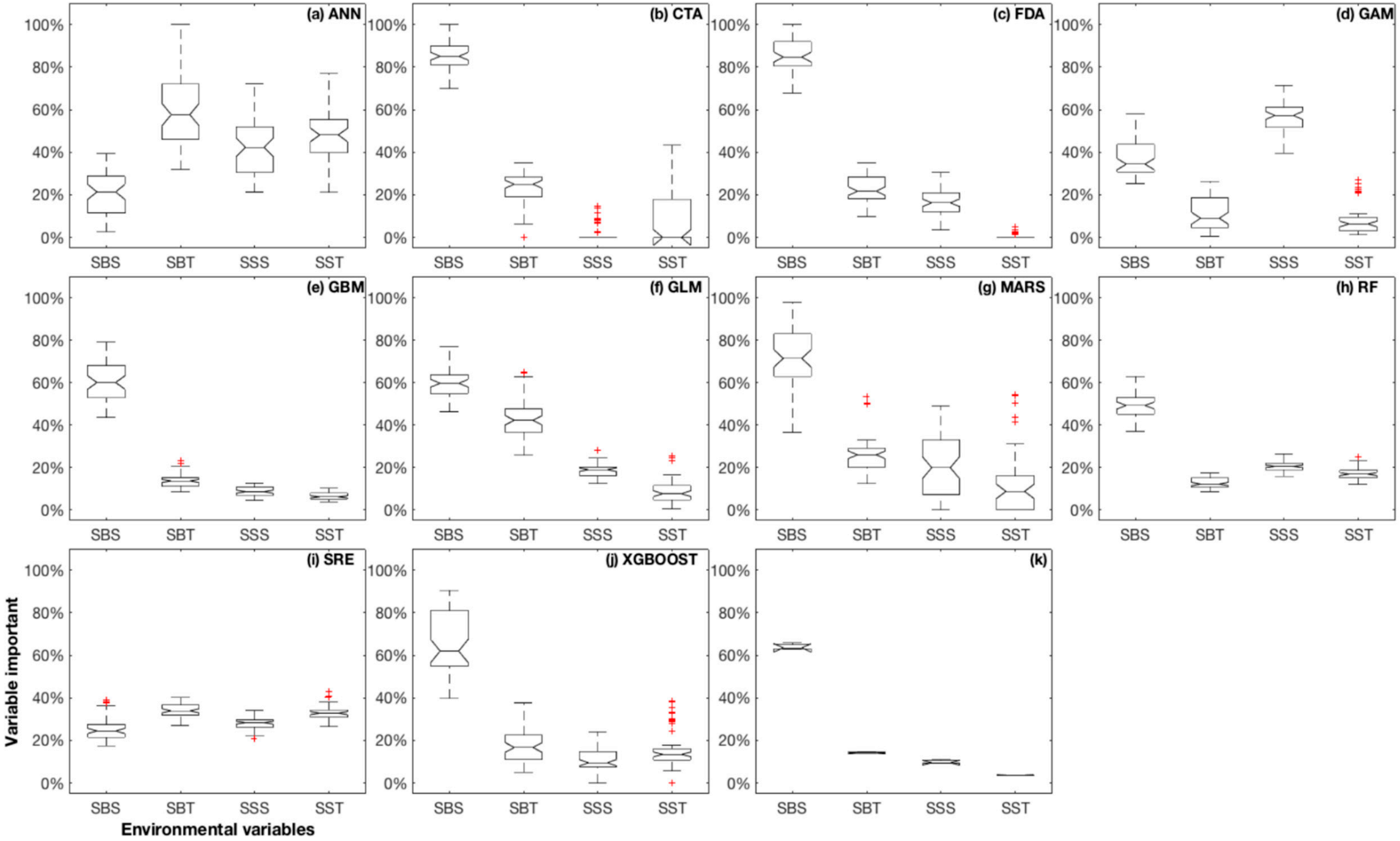

Figure S2. Box plots of the importance of environmental variables, including sea bottom salinity (SBS), sea bottom temperature (SBT), sea surface salinity (SSS), and sea surface temperature (SST), in the (a) artificial neural network (ANN), (b) classification tree analysis (CTA), (c) flexible discriminant analysis (FDA), (d) generalized additive model (GAM), (e) generalized boosting model (GBM), (f) generalized linear model (GLM), (g) multiple adaptive regression splines (MARS), (h) random forest (RF), (i) surface range envelope (SRE), (j) extreme gradient boosting training (XGBOOST), and (k) ten ensemble models.

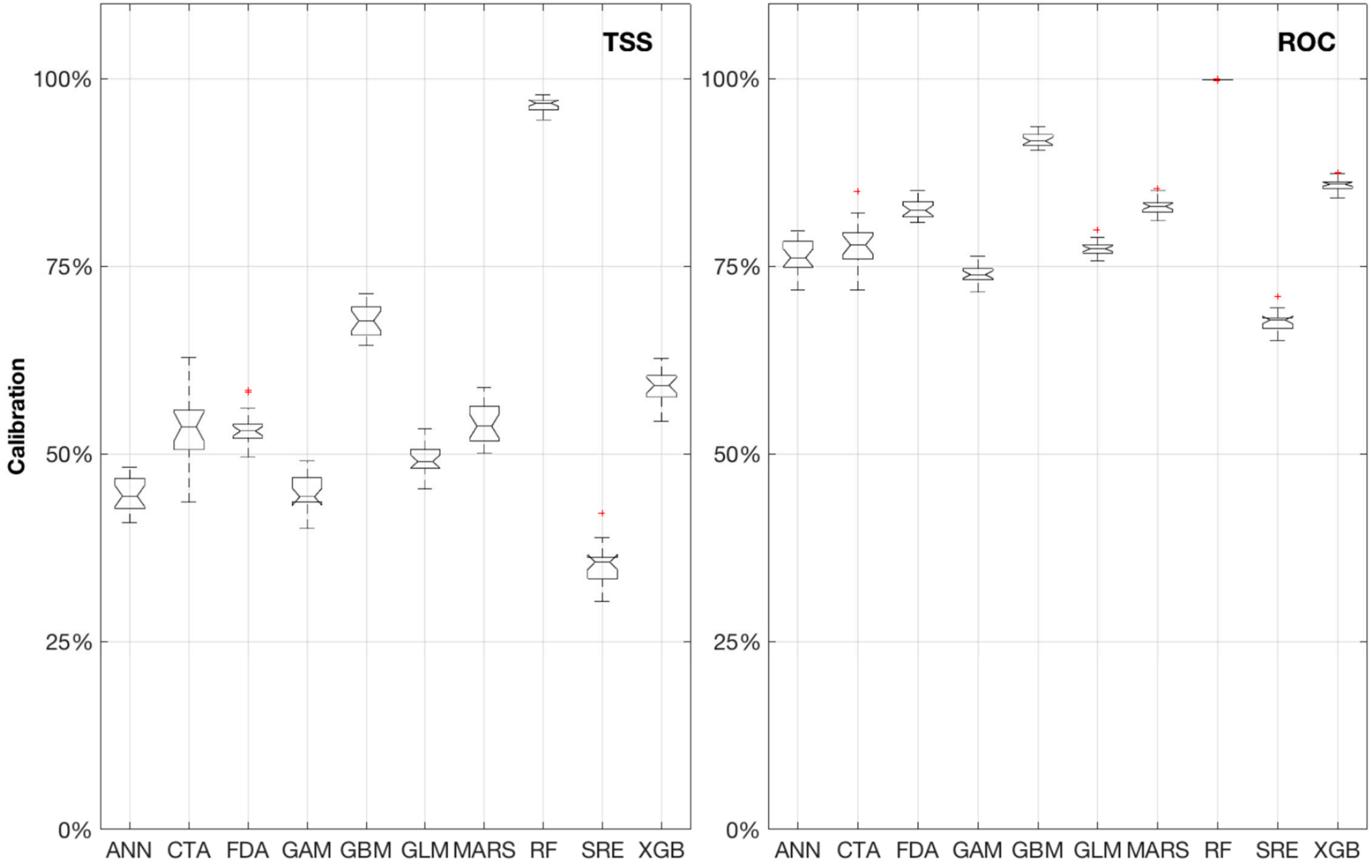

Figure S3. Calibration percentage (%) of TSS and ROC in the artificial neural network (ANN), classification tree analysis (CTA), flexible discriminant analysis (FDA), generalized additive model (GAM), generalized boosting model (GBM), generalized linear model (GLM), multiple adaptive regression splines (MARS), random forest (RF), surface range envelope (SRE), and extreme gradient boosting training (XGBOOST) methods.

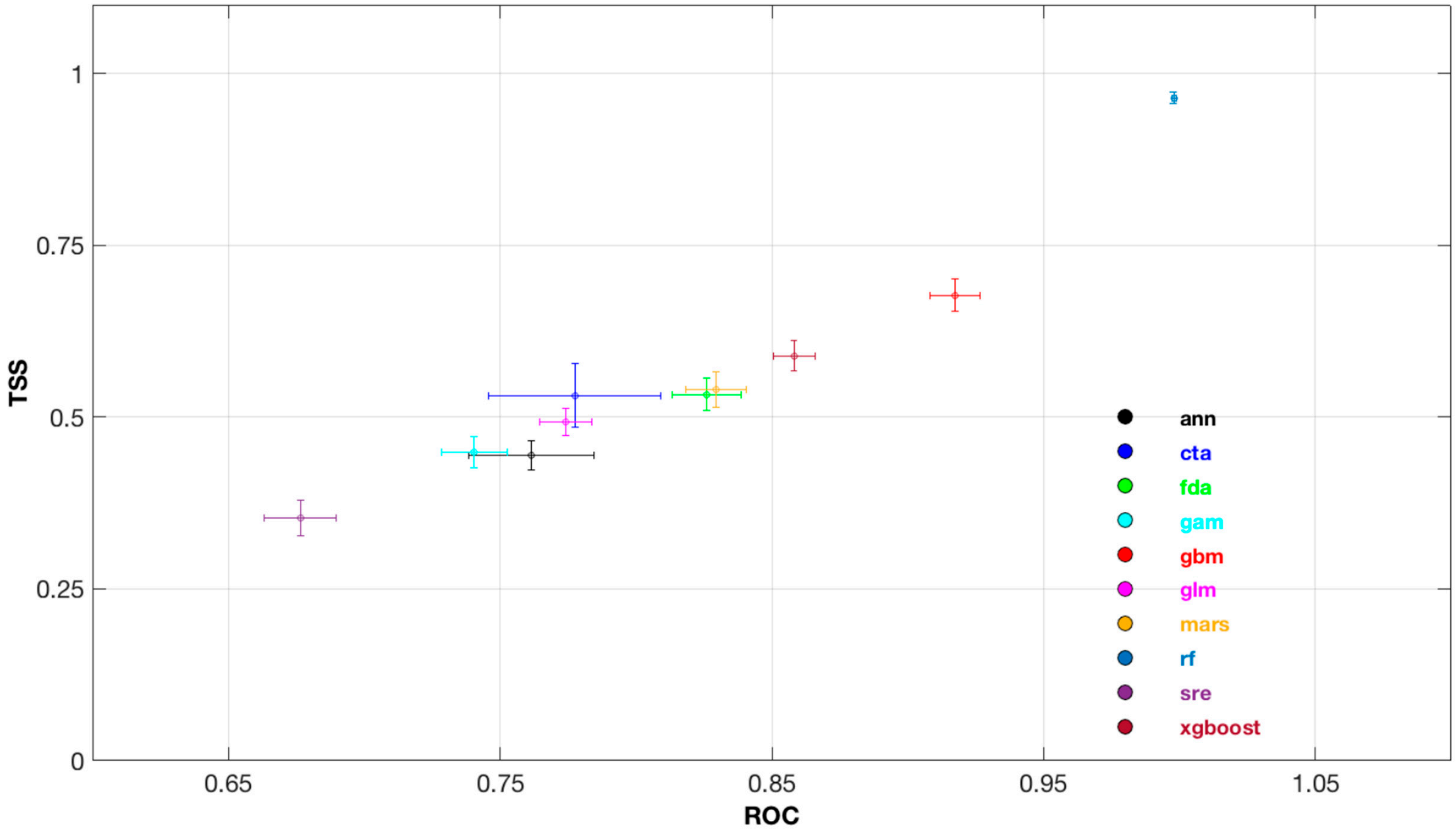

Figure S4. Ratio values of TSS vs. ROC with x-direction and y-direction error bars produced by the artificial neural network (ANN), classification tree analysis (CTA), flexible discriminant analysis (FDA), generalized additive model (GAM), generalized boosting model (GBM), generalized linear model (GLM), multiple adaptive regression splines (MARS), random forest (RF), surface range envelope (SRE), and extreme gradient boosting training (XGBOOST) methods, which are shown in black, blue, green, cyan, red, pink, dark yellow, dark blue, purple, and red-brown, respectively.

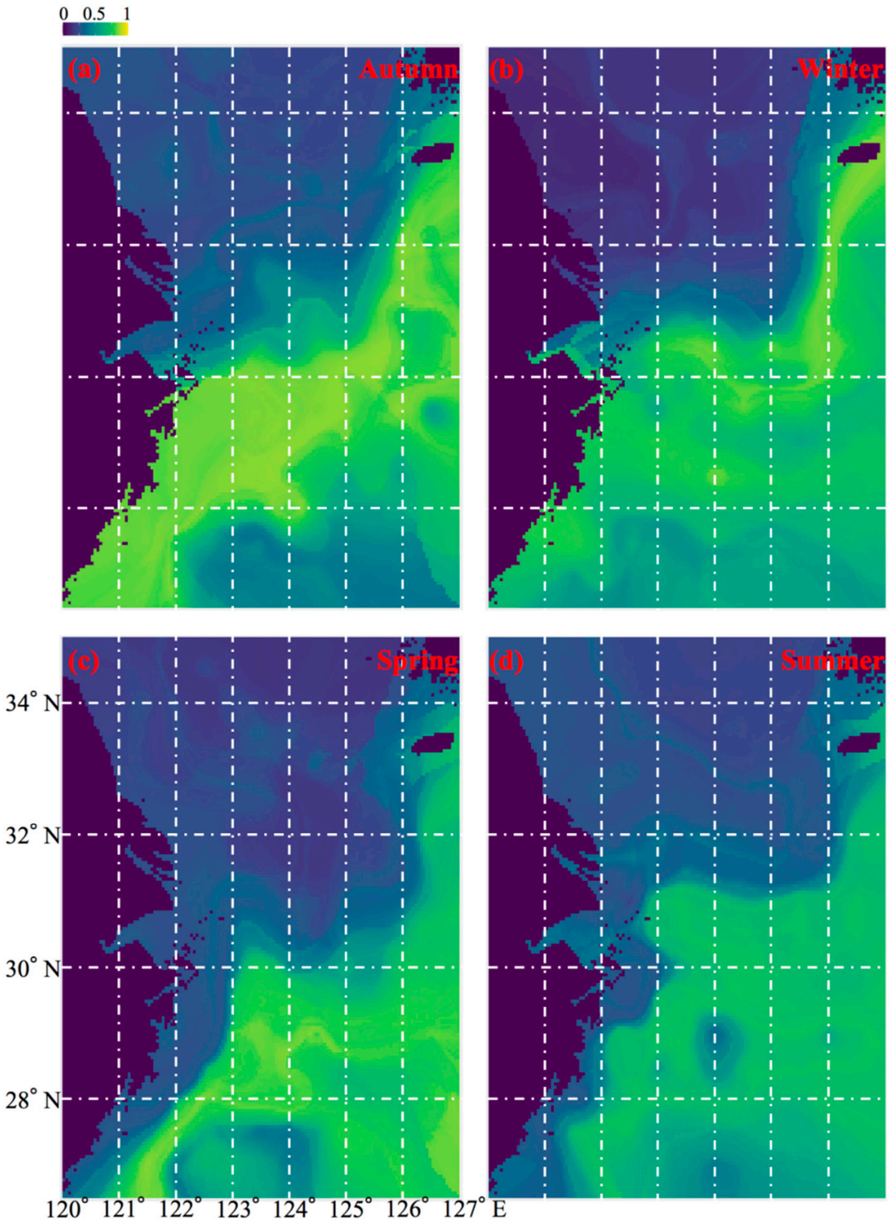

0
0.5
1
(a)
Autumn
(b)
Winter
(c)
Spring
(d)
Summer
34° N
32° N
30° N
28° N
120°
121°
122°
123°
124°
125°
126°
127° E

Figure S5. Spatial distribution patterns of *Abralia multihamata* in the study area predicted with the ensemble model consisting of the artificial neural network, classification tree analysis, flexible discriminant analysis, generalized additive model, generalized boosting model, generalized linear model, multiple adaptive regression splines, random forest, surface range envelope, and extreme gradient boosting training methods from spring to winter (a–d). The color change from blue to green indicates the range from low to high suitability.

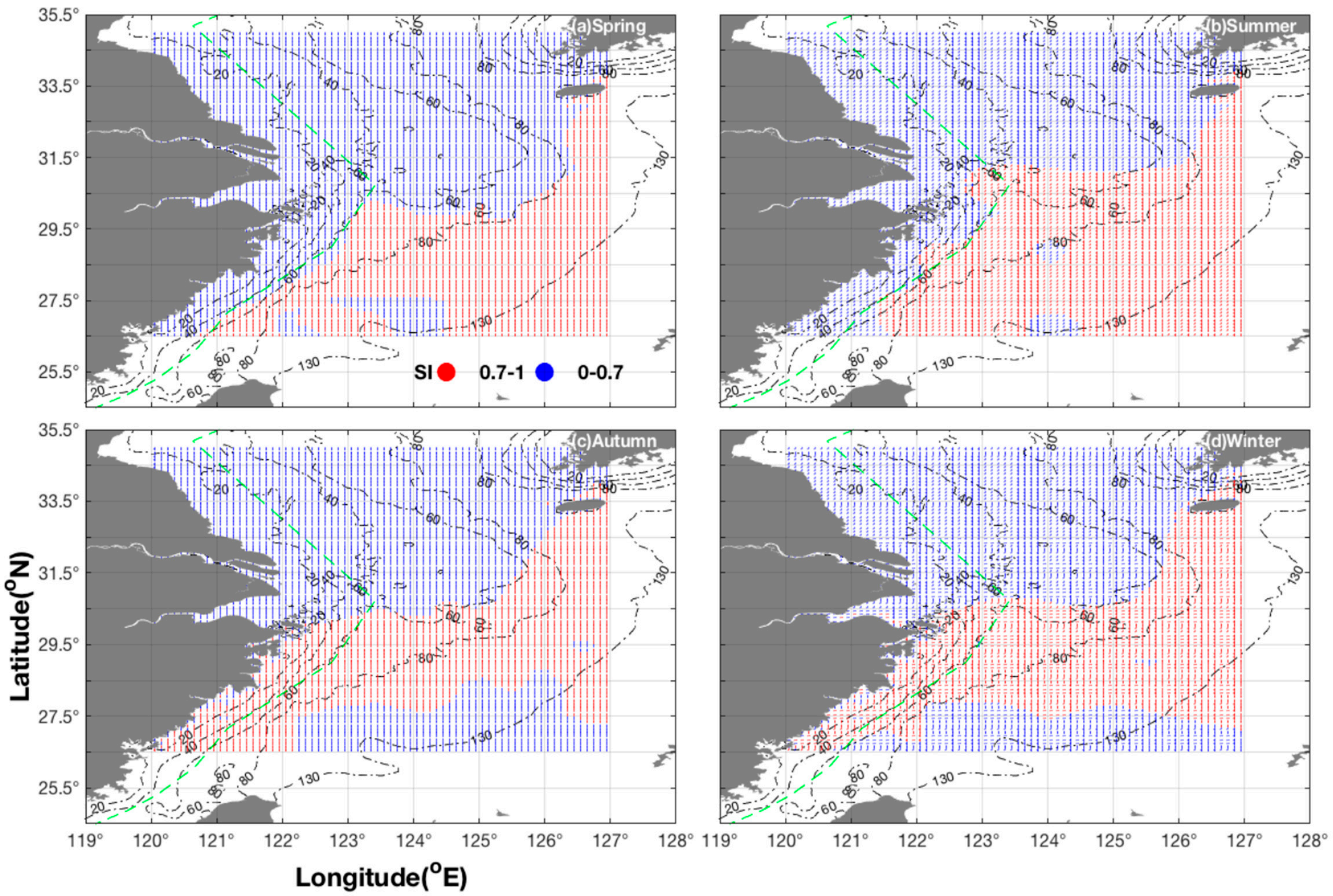

Figure S6. The predicted habitat suitability in different seasons (spring, summer, autumn, winter). The red and blue area indicate the suitability index of 0.7-1 and 0-0.7 independently.

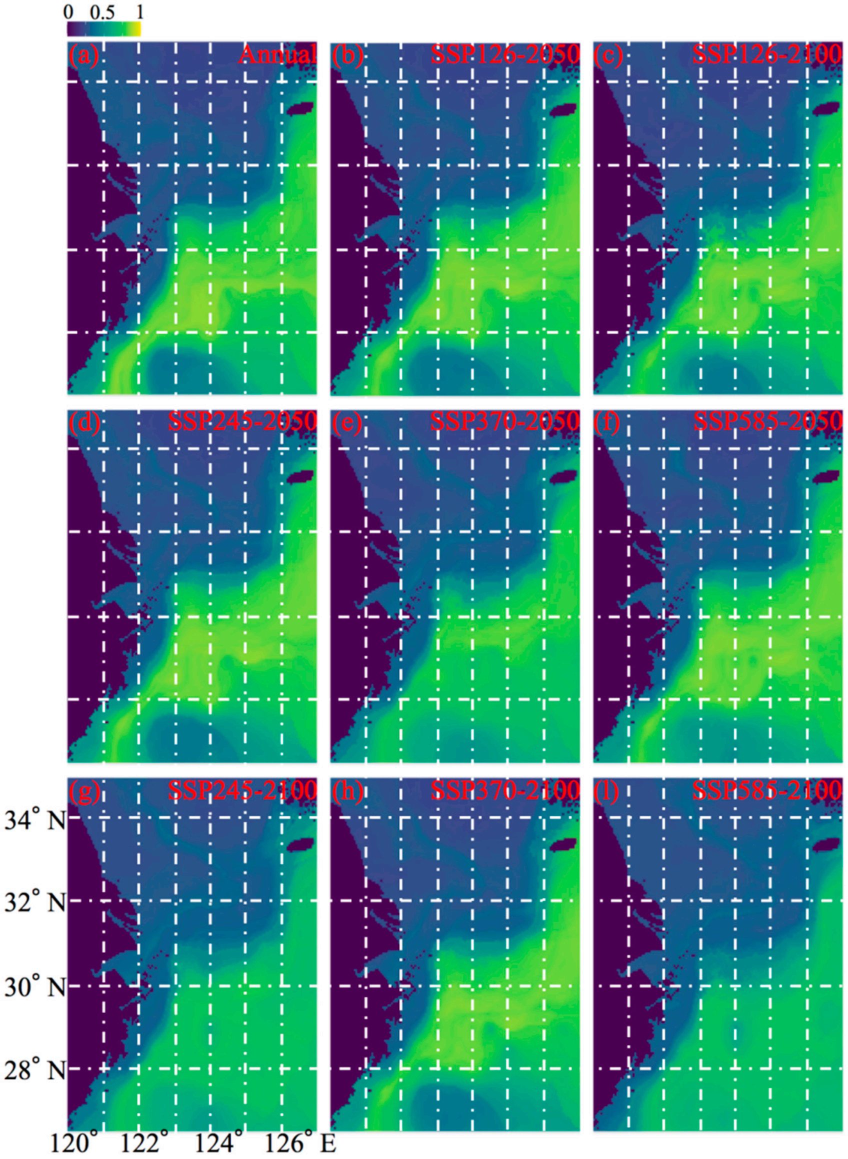
0
0.5
1
(a)
Annual
(b)
SSP126-2050
(c)
SSP126-2100
(d)
SSP245-2050
(e)
SSP370-2050
(f)
SSP585-2050
(g)
SSP245-2100
(h)
SSP370-2100
(i)
SSP585-2100
34° N
32° N
30° N
28° N
120°
122°
124°
126° E

Figure S7. Predicted spatial habitat distribution patterns of *Abralia multihamata* in the cases of (a) annual mean habitat; (b) SSP1-2.6 in 2050; (c) SSP1-2.6 in 2100; (d) SSP2-4.5 in 2050; (e) SSP3-7.0 in 2050; (f) SSP5-8.5 in 2050; (g) SSP2-4.5 in 2100; (h) SSP3-7.0 in 2100; and (i) SSP5-8.5 in 2100. The color change from blue to green indicates the range from low to high suitability.

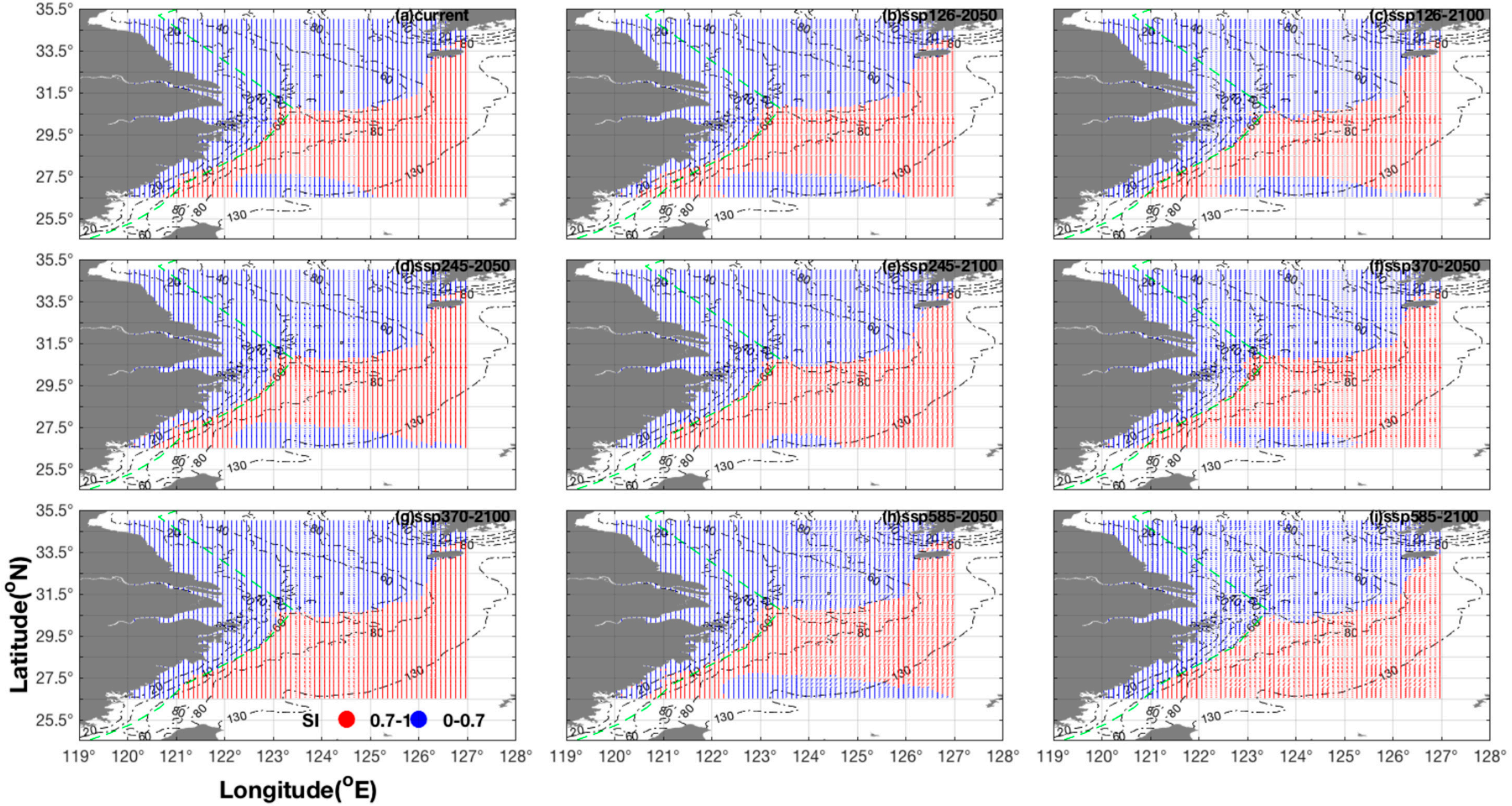

Figure S8. The predicted habitat suitability in different climate scenarios (a) current; (b) SSP1-2.6 in 2050; (c) SSP1-2.6 in 2100; (d) SSP2-4.5 in 2050; (e) SSP2-4.5 in 2100; (f) SSP3-7.0 in 2050; (g) SSP3-7.0 in 2100; (h) SSP5-8.5 in 2050; and (i) SSP5-8.5 in 2100. The red and blue area indicates the suitability index of 0.7-1 and 0-0.7 independently.
